# The IRONy in Athletic Performance

**DOI:** 10.3390/nu15234945

**Published:** 2023-11-28

**Authors:** William Kardasis, Ethan R. Naquin, Richa Garg, Tejas Arun, Jyotsna S. Gopianand, Eshani Karmakar, Jaya P. Gnana-Prakasam

**Affiliations:** School of Medicine, Saint Louis University, St. Louis, MO 63104, USA; william.kardasis@health.slu.edu (W.K.); enaquin22@stu.psm.edu (E.R.N.); richa.garg@health.slu.edu (R.G.); tejasarun9@gmail.com (T.A.); jyotsna.gopianand@gmail.com (J.S.G.)

**Keywords:** iron, exercise, athletes, iron deficiency, iron overload, diet, treatment, chelation

## Abstract

Iron is an essential micronutrient for athletes, intricately linked to their performance, by regulating cellular respiration and metabolism. Impaired iron levels in the body can significantly hinder athletic performance. The increased demand for iron due to exercise, coupled with potential dietary iron insufficiencies, particularly among endurance athletes, amplifies the risk of iron deficiency. Moreover, prolonged exercise can impact iron absorption, utilization, storage, and overall iron concentrations in an athlete. On the contrary, iron overload may initially lead to enhanced performance; however, chronic excess iron intake or underlying genetic conditions can lead to detrimental health consequences and may negatively impact athletic performance. Excess iron induces oxidative damage, not only compromising muscle function and recovery, but also affecting various tissues and organs in the body. This narrative review delineates the complex relationship between exercise and iron metabolism, and its profound effects on athletic performance. The article also provides guidance on managing iron intake through dietary adjustments, oral iron supplementation for performance enhancement in cases of deficiency, and strategies for addressing iron overload in athletes. Current research is focused on augmenting iron absorption by standardizing the route of administration while minimizing side effects. Additionally, there is ongoing work to identify inhibitors and activators that affect iron absorption, aiming to optimize the body’s iron levels from dietary sources, supplements, and chelators. In summary, by refining the athletic diet, considering the timing and dosage of iron supplements for deficiency, and implementing chelation therapies for iron overload, we can effectively enhance athletic performance and overall well-being.

## 1. Introduction

Iron, an essential mineral trace element (MTE), plays a pivotal role in energy metabolism, oxygen transport, and acid–base balance. Many metabolic enzymes are iron-dependent, including citric acid cycle enzymes aconitase and succinate dehydrogenase [1]. Iron is also indispensable for the electron transport chain governing adenosine triphosphate (ATP) production, as well as in gene regulation, cellular growth, and differentiation [2]. A profound understanding of the intricate mechanisms of iron homeostasis, storage, and regulation in the body is crucial to decipher its role in athletic performance.

Iron is a functional component of proteins involved in oxygen delivery (hemoglobin) and storage (myoglobin) [2]. Hemoglobin (Hb) is an iron-dependent protein that directly regulates the level of physical performance [1]. The levels of iron in hemoglobin and other iron-containing proteins within the body are significantly influenced by free iron levels from dietary intake and excretion patterns. Hepcidin, a peptide encoded by the *HAMP* gene, is one of the primary regulators of iron homeostasis in the body [3]. Hepcidin regulates iron levels by inhibiting ferroportin, a major transporter protein responsible for the transfer of dietary iron from the gastrointestinal tract into the blood [4]. Iron regulatory protein ferritin serves as an intracellular iron storage protein, while transferrin is a blood glycoprotein that binds to iron and mediates its transport to different parts of the body. Previous reports indicate that most individuals maintain normal levels of iron and iron-dependent proteins; however, athletes often have higher requirements. As an example, conventional ferritin levels are around 30 mcg/l, yet athletes, especially those engaged in high-altitude training where oxygen content in the air is lower, should have levels closer to 50 mcg/l [5]. Recent research has underscored the reciprocal relationship between iron and exercise performance, indicating that iron can impact athletic performance, and exercise in turn can affect iron levels [6]. Of particular interest is the observation that exercise can lead to a condition known as iron deficiency non-anemia (IDNA). This phenomenon is paradoxical as IDNA can also compromise athletic performance [7].

Standard hemoglobin levels are 14 g/dL for men and 12 g/dL for women [1]. In a healthy individual, transferrin has an iron saturation range of 20–50%. On the contrary, transferrin saturation less than 20% indicates iron deficiency and more than 45% suggests iron overload. Normal serum ferritin values are, generally, 30–300 ng/L for men, and 30–200 ng/L for menstruating women. IDNA is characterized by low ferritin levels (<30 ng/mL in the absence of an inflammatory condition and <100 ng/mL in the presence of inflammation) and low transferrin saturation (<20%) with normal hemoglobin levels (13.5–17.5 g/dL for men and 12–15 g/dL for menstruating women). On the other hand, iron deficiency anemia (IDA) is defined by low serum ferritin (<30 ng/mL without inflammation and <100 ng/mL with inflammation) and low transferrin saturation (<20%) in addition to low Hb levels (<13.5 g/dL for men and <12 g/dL for women) [8]. Conversely, conditions of iron overload such as hereditary hemochromatosis (HH) can also hinder athletic performance [9]. HH is a condition in which iron absorption from the gut is increased due to mutation in any of the iron regulatory proteins. In cases of acute iron toxicity, serum iron levels can reach moderate overload between 350 and 500 micrograms/dL, with levels above 500 micrograms/dL considered severely toxic. Iron overload is characterized by high serum ferritin levels (>300 ng/mL in men and >200 ng/mL in premenopausal women), which may be non-specific however, as inflammation, infection, or liver disease can also increase serum ferritin levels. Elevated transferrin saturation (>45%) can assist in further clinical diagnosis. Hence, HH is often associated with elevated serum ferritin and transferrin saturation [10,11].

Studies on the impact of HH on athletic performance are ambiguous. Some studies suggest that athletes with iron levels below the threshold of toxicity may exhibit enhanced athletic performance [12], while others indicate a reduction in athletic capabilities [9,13]. Nonetheless, it is well established that untreated HH can lead to organ damage, including the heart, skeletal muscles, and liver [8,14,15,16]. Furthermore, excess iron can induce zinc deficiency, which can further limit exercise performance as zinc also plays a vital role in various biological processes in the body [1].

Built upon the literature evidence, this narrative review aims to provide a comprehensive understanding of how disturbances in iron homeostasis can affect athletic performance. This includes iron deficiency, a well-documented issue among athletes, and iron overload, which has received less attention in the context of athletic performance. Given that athletes, both professional and amateur, are often targeted by the nutritional supplement industry with the assurance of improved performance, it is crucial to investigate the relative advantages of iron supplementation, and simultaneously, the risks associated with iron overload. Additionally, this review seeks to explore the treatment options for both iron deficiency and iron overload including the optimal scheduling of oral iron therapy, dietary factors that may enhance or hinder iron absorption, and therapies used to manage iron overload and toxicity. The goal of this review is to provide insights into promoting holistic well-being in athletes to achieve their optimal performance.

## 2. Methodology

A literature search was conducted using the online electronic databases PubMed and Science Direct by searching the keywords ‘iron’, ‘athlete’, ‘iron deficiency’, ‘iron overload’, ‘exercise’, and ‘physical activity’. This search included human studies and animal experiments. Randomized controlled trials (RCTs), experimental studies, and reviews were included. Articles within the last 15 years were prioritized; however, earlier articles were also included to provide relevant background information. In our screening, we excluded duplicate articles, articles not in English, and articles with no full text available. We excluded articles that were not relevant to answering our research questions (like RCTs on humans not correlating athletic performance and iron status, RCTs on athletes with other conditions that influenced iron status, and RCTs on humans including participants who were non-athletes). Information from these articles was synthesized together to create a narrative review of the literature. The authors have no financial or non-financial conflicts of interest that influenced the selection of research articles, or the outcomes and interpretations in this review.

## 3. Exercise Leads to Iron Deficiency

Exercise, particularly high-intensity and endurance training, can result in a substantial depletion of the body’s iron stores with reductions of up to 70% observed when compared to the general population. Athletes undergoing intense training frequently encounter an elevated risk of iron deficiency with or without anemia. This condition is often linked to a combination of factors, which paradoxically can also negatively impact athletic performance [7,17]. The mechanisms underlying this phenomenon are diverse, including poor dietary choices, increased iron requirements, elevated iron loss, inflammation, foot strike hemolysis, thermochemolytic sweat loss, exercise-induced ischemia leading to gastrointestinal iron loss, hematuria, and decreased iron absorption due to exercise-induced surges in hepcidin mediated by interleukin 6 (IL-6) [5,7,17]. It has been found that sweat may contribute up to 22.5 μg of iron lost per liter of sweat [18]. Runners are especially vulnerable to hematuria, due to running-induced trauma in the posterior wall of the bladder [19].

Notably, IDNA is more prevalent in female athletes, affecting approximately 15–35% of female athletes compared to 5–11% of male athletes [20]. Hormones also play a significant role in the etiology of IDNA in athletes. Large training loads can suppress gonadotropin-releasing hormone (GnRH), along with a decrease in luteinizing hormone (LH) and follicle-stimulating hormone (FSH), which in turn leads to decreased estrogen (E1) levels in women. Additionally, estradiol (E2) supplementation has been found to downregulate hepcidin production. Consequently, lower estrogen may correlate with higher hepcidin levels, in turn, impairing iron absorption in the gastrointestinal tract. Furthermore, extreme acute endurance exercises, such as Ironman competitions, can reduce testosterone levels, which, in turn, suppress hepcidin, further influencing iron levels [20].

In some cases, the hematological conditions observed in athletes may differ slightly from IDNA and present symptoms similar to anemia. As an adaptation to regular aerobic exercise, plasma volume can increase markedly while erythrocyte volume increases mildly, leading to lower Hb levels due to dilution, manifesting a condition known as pseudo-anemia, or sports anemia [21]. Sports anemia is characterized by increased erythrocyte destruction, impaired iron absorption, and gastrointestinal (GI) blood loss [1]. Possible mechanisms for the development of sports anemia include GI tract malfunction due to an increase in sympathetic tone secondary to intense exercise or stress-induced mechanical destruction of hemoglobin [22]. Other adaptations to training include decreased bone marrow iron stores and increased iron absorption in elite distance runners. However, these low iron levels may be transient, as one study revealed that the initial drop in iron, hemoglobin, and hematocrit levels in female cross-country runners is typically restored to normal over time [1].

Recent research suggests that the type of training an athlete engages in influences the type and severity of blood-related complications. For example, one study found that endurance athletes tend to have lower hemoglobin and hematocrit levels compared to strength and mixed-trained athletes, potentially due to exercise-induced plasma-volume expansion [23]. Runners often exhibit lower haptoglobin levels, which may be attributed to the trauma involved in running, triggering red blood cell destruction. Decreased ferritin levels have been observed in all athletes, with the most pronounced reductions seen in runners [23]. Furthermore, intense training can result in the absorption of iron without adequate binding to transferrin, leading to the release of free iron, which can catalyze reactions producing harmful free radicals. This condition, known as overtraining syndrome, is explored less than IDNA, but can potentially be more dangerous [2].

## 4. Iron Deficiency Impairs Athletic Performance

The relationship between iron status and physical activity is complex and bidirectional, as both influence each other. For instance, research involving iron-deficient rowing athletes demonstrated that lower ferritin stores were linked to slower rowing ergometer time trial performance [20]. Similarly, experiments conducted with animal models that were fed a low iron diet (LID) revealed lower maximum oxygen uptake and increased muscle fatigue. This was corroborated by a 55% reduction in the respiratory capacity of muscle homogenates in the low-iron group [24].

Several mechanisms may account for this decline in athletic performance in iron-deficient individuals. In the aforementioned mice study, low iron diet (LID) groups exhibited lower levels of hemoglobin, cytochrome c, cytochrome oxidase, and mitochondrial glycerol-3-phosphate dehydrogenase activity, potentially leading to impaired cellular respiration and metabolism. Additionally, reduced oxygen availability in iron-deficient individuals may necessitate greater reliance on anaerobic metabolism, resulting in elevated lactate concentration, lower blood pH, and depletion of muscle glycogen. IDNA also leads to decreased mitochondria, muscle activities of myoglobin, succinate dehydrogenase, and cytochrome c, reinforcing the connection between iron deficiency and reduced exercise capacity [24]. Notably, another study utilizing mice models revealed that iron deficiency impaired overall growth, a condition that was ameliorated upon iron repletion [25]. Hb concentrations were significantly lower in iron-deficient groups, with a notable redistribution of hemoglobin to the brain during exercise, implying reduced availability in muscles. This group also exhibited reduced glycogen levels, further exacerbating the impact of iron deficiency on physical performance [25]. Since iron is needed for oxygen transport via hemoglobin and serves as a cofactor for several enzymes involved in the aerobic metabolism pathway, these underlying biochemical mechanisms cause low iron to hinder optimal athletic performance [26].

The recommended dietary intake of iron is 8 mg/day for males and 18 mg/day for premenopausal females. However, these recommendations do not consider the augmented iron demands attributable to exercise. Therefore, it is imperative to recognize that athletes, particularly endurance athletes like runners, may need 70% more iron intake per day [27]. Female athletes often need additional iron supplementation due to menstrual blood loss [28]. Moreover, IDNA is at least twice as common as iron deficiency anemia (IDA), yet it often goes unnoticed by clinicians [29]. IDA is well reported to cause symptoms such as fatigue, muscle weakness, and compromised cognitive function [30], all of which can contribute to poor athletic performance. In many instances, early detection of IDNA is important, especially in high-risk patient groups. In a study of 121 recreationally active adults, IDNA was prevalent in 29% of females, compared to 4% in males. Using the transferrin receptor–ferritin index, these numbers increased to 36% for females and 6% for males [31]. In another study of 14 female runners, 50% were found to be iron deficient at baseline, with over 70% deficient after a training regimen [32]. Various factors elevate the risk of iron deficiency, including vegan or vegetarian diets, frequent blood donation, adolescence, eating disorders such as anorexia nervosa or bulimia nervosa, heavy menstruation, gastrointestinal disorders or surgeries, and intensive athletic training [5].

IDNA remains a diagnostic challenge, lacking well-defined criteria. Suspicion of IDNA should arise when a patient exhibits symptoms of iron deficiency without anemia, combined with low serum ferritin levels [33]. A thorough medical history is also crucial, considering factors such as blood donations, accidents resulting in significant blood loss, or surgery, as well as a diagnosis or family history of celiac disease or atrophic gastritis. Diagnosis can be particularly difficult when trying to distinguish IDNA from an infection. Infections that can cause a decrease in iron content include malaria, hookworm, and HIV. There are also a variety of drugs that can cause a decrease in iron and may be confused with IDNA in an athlete, including many antibiotics, tuberculostatic, and antiretroviral drugs. However, many of these causes may in fact present as IDA (microcytic) or macrocytic megaloblastic anemia, with mean corpuscular volume (MCV) as the distinguishing diagnostic factor [34].

In summary, iron deficiency can significantly impair athletic performance. When considering this finding in conjunction with the knowledge of exercise-induced iron deficiency, it appears paradoxical indicating that exercise can lower an individual’s iron stores, subsequently reducing their ability to exercise effectively. However, deficiency is not the only iron-related issue with the potential to interfere with exercise capacity.

## 5. Iron Overload and Athletic Performance

While the impact of iron deficiency on athletic performance is well documented, the consequences of iron overload have received less attention. Iron overload is a condition where excess iron accumulates in the body, potentially leading to oxidative stress and cellular damage in various tissues. Athletes with iron overload due to HH have shown improvement in athletic performance when the iron levels are below the toxic threshold. However, the long-term risks associated with iron overload may eventually cause compromised athletic capabilities. This risk extends to HFE and non-HFE genetic hemochromatosis as well as secondary hemochromatosis (or non-hereditary iron overload). Non-HFE genetic hemochromatosis may be caused by a mutation in one of the other iron regulatory proteins like hemojuvelin (HJV), transferrin receptor 2 (TFR2), hepcidin (HAMP), or ferroportin (SLC40A1) [10]. The unique geographical distribution and inheritance pattern for HFE and non-HFE hemochromatosis are shown in Table 1. Athletes who have blood relatives diagnosed with HH are recommended to undergo preventive genetic screening [10,11].

Iron overload may be from iron-loading anemias or from a non-genetic cause, such as transfusional iron overload secondary to sickle cell disease or thalassemia major [7,11]. It can also be caused by dietary intake of iron-rich foods like red meat or long-term oral iron supplementation [35]. High serum ferritin levels caused by inflammation, alcoholic hepatic steatosis, nonalcoholic steatohepatitis, or hepatitis B or C infection should also be considered in the differential diagnosis of non-genetic causes of iron overload [36,37].

In cases of acute iron toxicity, patients may progress from gastrointestinal symptoms such as abdominal pain, vomiting, diarrhea, and passing blood in the urine and stool, to severe symptoms such as elevated alanine aminotransferase (ALT) levels and the risk of liver failure [38]. Chronic iron overload induces the production of free radicals, which accumulate in muscles, causing inflammation and damage. Iron accumulation has been shown to impede myogenesis through oxidative stress, ultimately resulting in skeletal muscle atrophy via ubiquitin ligase-dependent pathways [39,40]. This leads to extended recovery times and decreased muscle strength, both of which may affect an athlete’s performance [41]. A recent study based on 100 athletes participating in various sports demonstrated that those at higher risk for iron overload cycled faster and displayed greater oxygen-carrying capacity. This suggests that athletes with elevated risk, often associated with genetic variations (most commonly a mutation in the homeostatic iron regulator-HFE gene) experience less fatigue, improved oxygen supply to the muscles, and enhanced post-exercise recovery [12]. Therefore, iron supplementation should be approached with caution in such cases.

There are several underlying mechanisms that may contribute to a decline in athletic performance in chronically iron-overloaded individuals. Iron overload is associated with oxidative stress-mediated damage to various systems including the cardiovascular system [42]. Chronic iron overload inhibits the beneficial effects of aerobic exercise on the vasculature by hindering endogenous antioxidant responses and anticontractile effects mediated by nitric oxide bioavailability [43]. Oxidative stress is shown to impair mitochondrial function, including the regulation of cytochrome C phosphorylation and respirasome factors, contributing to mitochondrial dysfunction [44,45], further impairing cellular respiration and metabolism [46,47]. Moreover, the elevated lactate concentration in the high iron diet group suggests a heightened reliance on anaerobic metabolism due to mitochondrial dysfunction, in turn leading to reduced blood pH and depletion of muscle glycogen [48]. Besides maintaining cellular energy, studies extensively highlight the role of mitochondria in skeletal muscle atrophy [49]. Further, perturbation in iron homeostasis causes an overall decline in mitochondrial function as evidenced by diminished mitochondrial Ca^2+^ handling capacity, increase in mitochondrial oxidative damage, reduced succinate dehydrogenase and cytochrome-c levels, and impaired myoglobin activity in the muscles [36,50,51,52,53].

**Figure 1:**
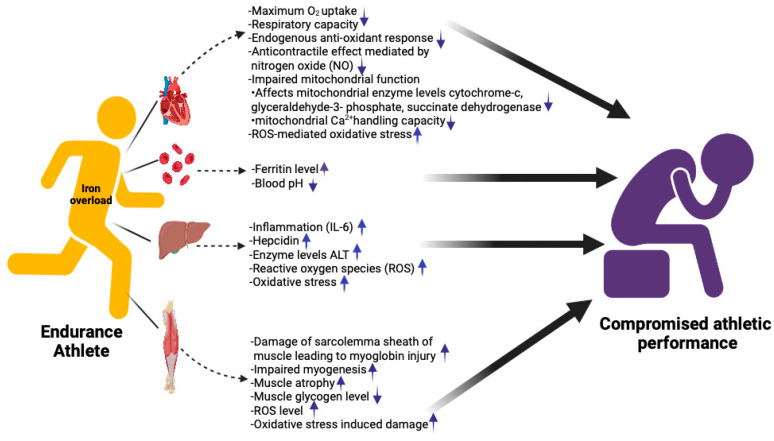
A schematic illustration outlining the potential factors contributing to compromised athletic performance as a result of excess iron accumulation in the body (created with BioRender.com). Up arrows indicate increase and down arrows indicate decrease.

Elevated serum ferritin correlates to increased body iron content and also serves as an inflammatory disease marker, indicative of cell damage [53,54]. A study involving 1000 male professional road cyclists found that more than 45% had ferritin levels over 300 ng/L, with 25% exceeding 500 ng/mL, with an increased predominance in older cyclists than in the younger ones. Three years later, those numbers dropped, but the elevated levels were linked to transient increases in liver enzyme alanine amino transferase (ALT), attributed to “heavy training”. Similar to complications described in hemochromatosis (HH), cyclists with increased ferritin levels are likely at a higher risk for developing cirrhosis, cancer, cardiovascular, and neurodegenerative diseases, which could impair long-term performance [55]. The induction of acute exercise-related inflammation, leading to potential liver dysfunction, can exert detrimental effects on athletic performance. Therefore, maintaining appropriate levels of liver enzymes is imperative to shield cells from inflammation [56]. In a study reminiscent of investigations related to iron deficiency, researchers examined a cohort of iron-overloaded athletes. The findings revealed that 15% of male athletes and 4.7% of female athletes in the study met the criteria for iron overload, suggesting that iron overload might be prevalent, particularly among male recreational marathon runners [57]. Hence, iron overload, especially when uncontrolled or unmanaged, may impair athletic performance, and pose challenges for professional athletes. Excessive iron levels, akin to deficiency, can disrupt the exercise capacity of the athletes through various causal relationships in the body as shown in Figure 1. Consequently, individuals already at an increased risk of iron overload or having a high baseline iron status should refrain from additional iron supplementation. Further research in athletes is warranted to gain a comprehensive understanding of the acute and long-term implications of iron overload throughout an athlete’s career. To prevent long-term complications, all cases of iron overload should be managed appropriately in clinical settings, as discussed later in this article.

**Figure 2:**
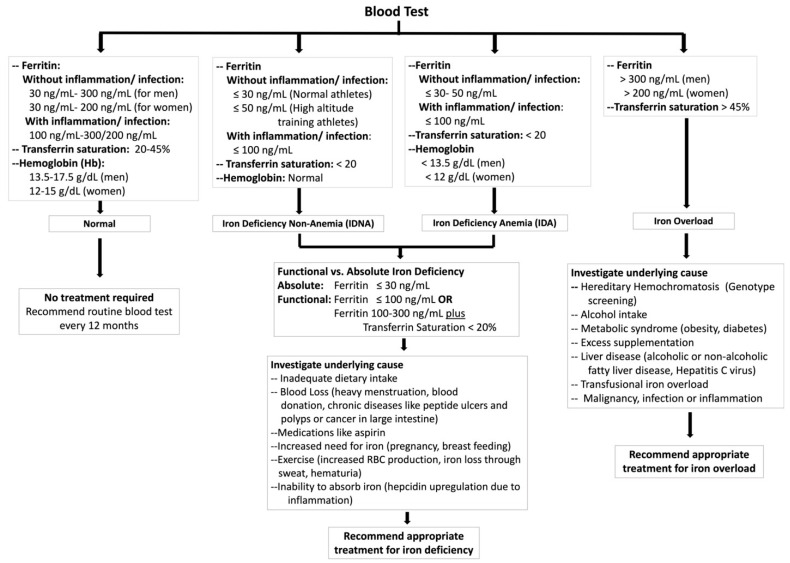
Algorithm for diagnosing iron deficiency and iron overload in adult athletes based on current evidence [10,11,58,59,60].

Many athletes, regardless of their baseline iron status, resort to iron supplementation in pursuit of a competitive edge. Athletes must pay close attention to their iron status, whether it leans toward deficiency or overload, to optimize their performance and safeguard their long-term health. An algorithm for diagnosing iron deficiency and iron overload in adult athletes is shown in Figure 2.

## 6. Treatments and Solutions

The primary approaches to address iron deficiency or overload typically involve dietary counseling, oral iron repletion, or the utilization of iron chelators. Intravenous (IV) or intramuscular (IM) iron repletion is generally not recommended unless initial treatment steps such as counseling and oral repletion prove ineffective or when managing a concurrent medical condition [5]. Furthermore, iron absorption is influenced by a multitude of activators and inhibitors, rendering iron management a complex task. In this section, we will explore various strategies aimed at maintaining optimal iron levels to enhance an athlete’s performance.

### 6.1. Dietary Counseling

Dietary iron is available in two forms: heme or non-heme. Heme iron is absorbed more efficiently, with a 25% absorption rate, in contrast to 17% observed for non-heme iron (Table 2). Eating high-iron foods derived from animal sources, such as meat, poultry, and seafood, has a superior bioavailability as they are in the heme form of iron. Non-heme iron, from plant sources such as legumes, nuts, and spinach, has a lower bioavailability, which can complicate the treatment of IDNA in vegetarians and vegans through dietary counseling alone and may not be sufficient to correct the iron deficiency [61]. Additionally, several iron-binding ligands from our diet act as inhibitors or activators of iron absorption. Phytates and polyphenols found in plants inhibit iron absorption by forming a metal complex with iron [62,63]. Tea, coffee, red wine, and cocoa are common beverages high in polyphenols. One study concluded that beverages high in polyphenols can inhibit the absorption of dietary non-heme iron robustly. It showed that 20–50 mg of polyphenols per serving reduced the non-heme iron absorption following a bread meal by 50–70%, with the reduction being 60–90% for 100–400 mg of polyphenols per serving, compared to a water control. Calcium is also shown to have an inhibitory effect on iron absorption, affecting both heme and non-heme iron absorption [64], although through a mechanism not fully elucidated. Benkhedda et al. showed that when treating women with marginal iron status, administering 500 mg calcium carbonate showed a significant reduction in iron absorption from a single meal from 10.2% to 4.8% [65]. In a study of 788 school children aged 6–11 years old, the authors compared the effect of calcium on iron absorption, both with and without vitamin C added, in a casein/whey-based drink that was fortified with ferrous sulfate. Participants included children with IDA and iron-replete conditions. The results showed that the addition of calcium showed a reduction in the mean iron absorption in the children by 18–27%, and the reduction was dose-dependent. As the dose of calcium increased, the absorption of iron decreased. Another mineral, zinc, was also found to have an acute inhibitory effect on iron absorption, which was hypothesized to occur due to competitive inhibition by zinc, as zinc and iron share similar mechanisms of uptake [66,67]. Therefore, it is advisable to contemplate a spaced-out approach when administering zinc and iron supplementation in populations experiencing deficiencies in both these essential minerals. In meals, however, this interaction is generally not seen [68].

In contrast to the inhibitors, Vitamin C (ascorbate) is well reported to aid in the absorption of iron, notably acting as an electron donor for ferrireductase activity in the duodenum to aid in the uptake through the divalent metal transporter-1 [69]. A study in recent years reported that vitamin C enhances iron uptake significantly from transferrin iron (Fe-Tf) in humans through a novel intracellular reductive pathway. The study suggests that ferrireduction is amplified in the endosomes containing transferrin, which then enhances iron mobilization from the endosome, ultimately leading to an increased iron delivery to cells [70]. There is also evidence in the literature supporting vitamin C’s ability to override the inhibitory effects of polyphenols, phytates, and calcium to improve iron absorption [71,72]. Indeed, when vitamin C was added to the drink in the study of calcium effect on iron absorption, the inhibitory effect of calcium was reversed by the addition of vitamin C in molar ratios of 2:1 and 4:1 [72], showing enhancers of iron absorption have the potential to override inhibitors of iron absorption. Therefore, athletes with iron deficiency can supplement their diets with vitamin C-rich foods, such as citrus fruits, tomatoes, strawberries, and leafy green vegetables [73]. However, a recent study found that in a mixed meal, dietary vitamin C is not enough to mitigate the inhibitory effects. Rather, the benefits of vitamin C on iron absorption are best seen when taking it alone with the iron supplement [74]. In addition to the inhibitors and activators, the timing of iron intake also affected its absorption. As discussed earlier, hepcidin levels influence iron absorption. Studies conducted by McCormick et al. compared the effect of exercise in the morning and afternoon on iron absorption from breakfast and dinner via traceable iron isotopes. In association with the diurnal increase in hepcidin concentration in the afternoon, the study demonstrated enhanced iron absorption in the morning. A recent report showed that oral iron absorption can be augmented in the morning without food or beverages such as coffee, which are high in polyphenols. It is recommended instead to have a beverage that is high in vitamin C. Taking a supplement with orange juice can equate to a ~4-fold increase in the absorption of iron [74].

### 6.2. Oral Iron Treatment

Oral iron repletion therapy remains the preferred, optimal, economical, and safest treatment. A study was conducted on 42 women with IDNA, given either oral 100 mg of ferrous sulfate daily or placebo for 6 weeks, and were subjected to training for 30 min per day and 5 days of the week. It was reported that supplementation increased serum ferritin in the experimental group compared to the placebo. The study also concluded that IDNA impairs favorable adaptation to aerobic exercise [7]. Another study by Hinton on athletes with IDNA concluded that supplementation with 30 mg ferrous sulfate over 6 weeks significantly improved endurance capacity [75]. In another study of 20 active women with low serum ferritin levels, participants were randomly assigned to a placebo or experimental group, with the experimental group receiving daily iron supplementation for 8 weeks. The study found that the experimental group’s VO_2max_ was significantly greater and the post-endurance blood lactate levels were decreased in the experimental group [76]. When considering fatigue, in a systematic review including 18 trials and 2 companion papers (enrolling 1170 patients total), it was seen that adults with IDNA experience reduced subjective measurements of fatigue in the presence of iron supplementation, but no significant objective improvement in physical performance [77]. Similarly, in another study, 18 female runners with IDNA were pair-matched and randomly assigned to either the iron supplementation or placebo group. The study concluded that although both the experimental and placebo groups increased their time to exhaustion, there was no significant difference between the groups and no significant difference in VO_2max_ results [78]. Thus, there remains a debate on whether IDNA affects the performance of athletes, as there is literature supporting both positions [79]. However, it is widely accepted that IDA does affect performance, and iron repletion consistently shows benefits.

In a study performed on 24 elite runners over a 3-week training camp at 2016 m altitude, it was concluded that a single 200 mg dose nightly elemental iron supplement was superior at increasing Hb_mass_ at altitude compared to a split-dose strategy of 100 mg twice per day (taken morning and evening). However, there was still a significant Hb_mass_ increase with the split dose strategy as well. It was noted that there was a greater incidence of GI symptoms with the single dose, although it subsided by the third week [80]. Comparing the administration of a once-daily versus split-dose strategy, Stoffel et al. 2017 concluded that there is no significant difference in the effectiveness of once-daily versus a split-dose strategy, measuring the total and fractional iron absorption (FIA) [81]. In a 2020 study of women with IDNA, it was shown that oral iron supplement therapy results in an increase in serum hepcidin that lasts for 24 h, which results in decreased iron absorption from other iron sources, such as supplements or dietary iron, for the 24 h period. It also found that the split-dose regimen showed a higher serum hepcidin concentration compared to the once-daily regimen [81]. However, there is a significant and growing body of research supporting alternate-day iron supplementation as opposed to daily or split-dosing regimens. Iron absorption was highest using alternate-day supplementation by measuring the fractional iron absorption (FIA), which showed 40–50% higher with the alternate-day measurement than the consecutive-day measurement. Additionally, the alternate-day dosing showed a lower rate of unwanted GI side effects, which can improve compliance in long-term treatment and result in better patient outcomes [81]. Between the two studies by Stoffel, it was concluded that iron supplementation as a single dose on alternate days is the optimal regimen for iron absorption. In a study applied to both male and female endurance runners with <50 ug/L serum ferritin, the daily-dose regimen was compared with an alternate-day dosing regimen for 8 weeks. It was shown that serum ferritin increased in both cohorts with comparable results, with a higher serum ferritin reported in males compared to females [82].

Iron can be supplemented in the form of inorganic salts, such as sulfates, or in the form of organic iron as complexes with amino acids or hydrolyzed protein. Usually, organic iron formulations such as ferrous gluconate exhibit higher bioavailability than inorganic forms like ferrous sulfate [83]. In addition, supplements that are formulated as ferrous (Fe^2+^) salts such as ferrous gluconate, ferrous sulfate, and ferrous fumarate are absorbed superiorly as compared to ferric (Fe^3+^) formulations [83]. Ferric formulations have an inferior bioavailability of about 3 to 4 times less than the ferrous formulations due to two reasons: [1] the iron must be converted from the ferric to ferrous state to be absorbed in the small intestine and [2] ferric iron has a poor solubility in alkaline environments [83]. Of the ferrous formulations, ferrous gluconate is more tolerated compared to the other two formulations and ferrous fumarate showed the least tolerability [84,85]. These supplements also contain varying amounts of elemental iron in them, which in turn determines their efficacy, as shown in Table 3. Approximately 10% of the oral elemental iron is absorbed by the body [58].

Promising research is being conducted in areas of improving iron formulations and delivery. Recent research on the development of microencapsulated iron and nanoparticulation further improves iron bioavailability. A study in 2019 of 558 IDA women showed that novel microencapsulated liposomal iron pyrophosphate had higher bioavailability and a more favorable palatability [86]. Another study conducted on nanoparticulation in 2022 in a rat model showed that ferric hydroxide-polyphosphate nanoparticles were a good source of bioavailable iron, in fact, greater by ~170% relative to ferrous sulfate [87]. In a 2020 study, vitamin D was found to influence post-exercise serum iron metabolism, with the vitamin D group having a lesser reduction in iron after their run, which led to the conclusion that vitamin D may prevent iron deficiency in athletes, particularly endurance athletes. The study also noted that vitamin D deficiency (25(OH)D < 30 ng/mL) and anemia often coexisted with each other [88].

### 6.3. Intramuscular Iron Treatment

Intramuscular (IM) injection of iron is rarely used owing to pain and soreness, as well as discoloration at the site of injection, which can be bothersome to athletes during training and performance. However, there is literature supporting that IM iron injections in IDNA women are significantly more effective in raising serum ferritin levels within 1 month compared to oral supplementation [89]. In a study of 15 elite female athletes with IDNA, the group that received 5 × 2 mL IM Ferrum H injections showed significantly increased serum ferritin levels compared to the placebo group as well as the baseline. Nonetheless, it did not result in an enhancement of physical performance, as evidenced by the selected physical tests conducted on the athletes in the study [90].

### 6.4. Intravenous Iron Treatment

Intravenous (IV) iron usage is generally indicated in specific scenarios, such as failure of dietary counseling and oral supplementation (intolerance or failure to restore normal levels) or cases of severe iron deficiency anemia. In athletes, IV administration of iron is particularly useful when a quick, rapid increase in iron stores is needed, or as noted previously, if the athlete cannot tolerate oral supplementation of iron due to GI side effects. In parenteral routes, there are also reports supporting both stances regarding IDNA’s effects on athletic performance. In a study by Burden et al. 2015, concluded that a single 500 mg IV iron dose in 15 runners with IDNA improves the iron deficiency for 4 weeks. However, it did not correlate to improved aerobic capacity [91]. Additionally, parenteral treatment causes side effects such as skin discoloration, headaches, joint pain, and in rare but severe cases, anaphylaxis [92], necessitating the need to consider a test dose. Some of the commonly used intravenous iron formulations and recommended dosages are shown in Table 4.

Identification and management of underlying causes of iron deficiency should be taken under consideration, especially if the patient does not respond to enteral iron supplementation. For example, GI blood loss or GI disorders, *H. pylori* infection, celiac disease, heavy menstruation, and eating disorders, among others, should be explored to improve therapy and address potentially serious conditions [5,92]. Medications that limit gastric acid secretion should also be taken into consideration such as cimetidine, omeprazole, or histamine H_2_-antagonists, as gastric acid is an important factor in absorbing iron, whereas the medication may impair absorption [97].

**Figure 3:**
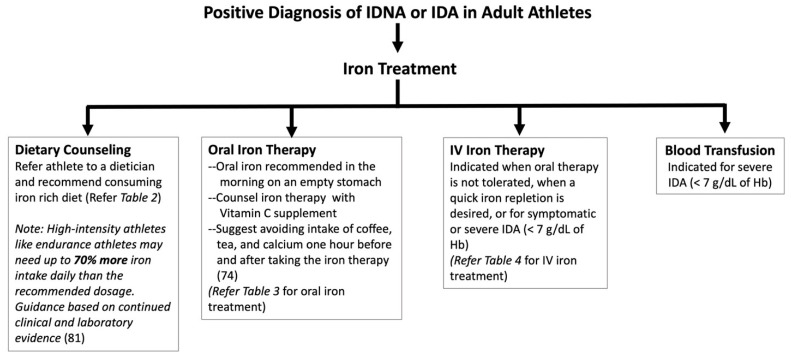
Algorithm for treatment of iron deficiency in adult athletes based on the current evidence [27,58,59,81,98]. Treatment choice recommended based on serum iron profile, underlying cause, and clinical judgment. Serum ferritin and transferrin saturation should be monitored regularly during and after treatment.

Overall, oral supplement of iron remains the safest and most cost-effective way to restore iron levels, although IM and IV are still relevant and beneficial for scenarios that require iron quickly, or for those who have difficulty tolerating oral administration. Recommendations on clinical management of iron deficiency are proposed in Figure 3.

### 6.5. Managing Iron Overload

In addition to managing iron deficiency, it is important to avoid excess iron or iron overload. In a study performed in 2005 on athletes, it was found that 30% of the 88 professional male cyclists and 14% of the 42 professional cross-country skiers had a serum ferritin over 350 ng/mL, a threshold value for iron overload. Zero percent of the 80 amateur road cyclists met the threshold, showing that the trend is generally present in professional athletes. Among all the subjects exceeding the 350 ng/mL threshold, none of the subjects were positive for HFE mutations for hereditary hemochromatosis (HH), ruling out hereditary causes, making secondary iron overload the main suspect [99]. Hence, iron supplementation should be generally discouraged unless indicated due to an iron deficiency diagnosed by a medical professional.

It is also important to establish if the cause of iron overload is primary or secondary. Primary iron overload can be caused by a dysregulation in the balance of iron, such as in hereditary hemochromatosis (HH). In HH, iron accumulation can occur in many organs, such as the liver, heart, and pancreas, among others. HH over time can manifest as cardiomyopathy, diabetes, or liver dysfunction. Because of advances in genetic testing, screening for HH has become easier. Secondary iron overload can result from excess iron consumption/intake, such as due to supplement intake, multiple blood transfusions, or iron-loading anemias [100]. As discussed earlier, excess iron can lead to long-term cellular damage through the formation of free radicals, leading to cell death, a process known as ferroptosis [42,101]. Another complication that can manifest is gastritis and ulcers [102,103,104,105]. There are also data that support a link between high iron and increased cancer risk [106].

**Figure 4:**
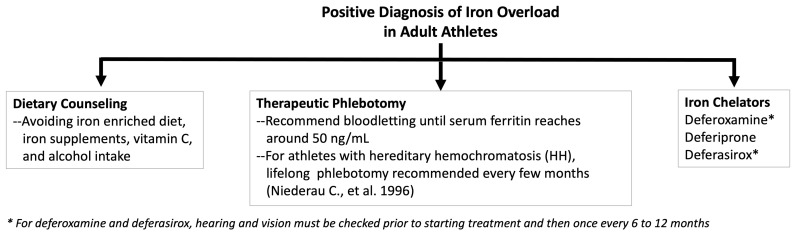
Algorithm for treatment of iron overload in adult athletes based on the current evidence [107,108,109,112]. Treatment choice recommended based on serum iron profile, underlying cause, and clinical judgment. Serum ferritin and transferrin saturation should be monitored regularly during and after treatment. Recommend cardiac T2* measurement by MRI every 6–24 months depending on the clinical risk for cardiac iron deposition [112].

For HH treatment, phlebotomy is the first line of therapy to reduce the accumulated iron in the patient [42], and phlebotomy therapy early on can result in a normal expected lifespan in patients [107]. It improves the liver and heart function and reduces the risk of liver cirrhosis and fatigue. Bloodletting is recommended 1 unit once per week (500 mL or 1 pint) and can be extended to every other week until serum ferritin reaches around 50 ng/mL [108,109]. For athletes with hereditary hemochromatosis, lifelong phlebotomy is recommended every few months [108]. Serum iron profile is tested every month (four sessions) initially, and then every 1 to 2 draws after serum ferritin is <200 ng/mL [110]. Synthetic iron chelators, such as deferiprone, deferasirox, or deferoxamine, are clinically used to treat secondary iron overload as well as primary overload in HH patients who do not respond to phlebotomy [110]. Natural compounds derived from spices and plants have also been investigated for their therapeutic iron-chelating properties. Curcumin, phytic acid in soy, quercetin found in red wine, green tea, apples, and berries, epigallocatechin-3-gallate (EGCG) in green tea, and tannic acid found in gallnut, wine, and tea have been reported to have iron-chelating properties [111]. However, herbal usage for iron chelation should be undertaken with caution as there is no sufficient research on the safe effective dosage and organ toxicity. In addition to therapeutic treatments, dietary modifications, and lifestyle changes by restricting alcohol, iron, and vitamin C supplement intake, are also beneficial in maintaining blood iron levels. Recommendations on clinical management of iron overload are proposed in Figure 4.

## 7. Conclusions and Future Directions

Iron is an essential component of our diet, playing a crucial role in many metabolic functions within the body. When the balance of iron is disrupted, it can have adverse effects on the body, leading to suboptimal athletic performance. Iron deficiency has been well documented in athletes, especially in endurance athletes. Intense exercise can lead to iron deficiency due to foot strike hemolysis, iron loss in sweat, hematuria, and decreased iron absorption. Paradoxically, iron deficiency impairs athletic performance further by lowering the levels of hemoglobin, cytochrome c, and cytochrome oxidase, in turn potentially hindering cellular respiration and metabolism. Conversely, iron overload may also lead to compromised athletic performance due to iron-induced oxidative stress and mitochondrial dysfunction. Serum ferritin levels and transferrin saturation provide valuable insights into the iron status of an athlete. However, inflammation or infection can also elevate serum ferritin levels, and hence clinicians must investigate all the potential underlying causes for an abnormal iron profile before any treatment is recommended.

Incorporating iron-rich foods is important in preventing iron deficiency. Furthermore, it is crucial to recognize that animal sources of iron contain the heme form, which has superior bioavailability compared to non-heme plant-based foods. Therefore, athletes, especially those at risk of iron deficiency, should be encouraged to optimize their dietary intake of iron. Additionally, individuals who abstain from meat products due to personal, health, or religious reasons may require additional support with oral supplementation. Special attention should also be directed toward other high-risk groups, such as females with heavy menstrual bleeding. Athletes should be encouraged to take vitamin C along with oral iron therapy while avoiding potential inhibitors of iron absorption, like tea, coffee, and milk, at least one hour before and after iron supplementation. Conversely, vigilance is warranted in monitoring iron repletion, to prevent the occurrence of iron overload and its detrimental consequences. In athletes with iron overload, phlebotomy is the frequently used therapy to mitigate the long-term risks associated with iron toxicity. In addition, iron chelation therapy and dietary restrictions are also considered for treating HH and transfusional iron overload secondary to conditions such as sickle cell disease and thalassemia major.

Further studies are essential to unravel the intricate relationship between iron status and athletic performance, particularly among athletes with iron deficiency without anemia. Additional research and innovation are imperative to alleviate the side effects of supplementation and enhance iron bioavailability. Similarly, in athletes with iron overload, a significant challenge lies in the development of a pharmaceutical agent that is both safe and practical, capable of reducing iron levels in a tissue-specific manner while preserving systemic iron homeostasis. Finally, improving diagnostic criteria for iron deficiencies and iron overload should be a focal point to enhance patient–physician health outcomes and improve athletic performance.

## Figures and Tables

**Table 1:** Hemochromatosis genes identified in the 1000 Genomes Project, Exome Sequencing Project, and Exome Aggregation Consortium datasets. Adapted from Girelli et al. 2022 [11].

Gene	Location	Geographical Distribution	Inheritance
*HFE* (p. Cys282Tyr)	Chromosome 6	Highest prevalence in Northern Europeans	Autosomal recessive
*HFE* (non-p.Cys282Tyr)	Chromosome 6	Highest prevalence in Northern Europeans	Autosomal recessive
*HJV*	Chromosome 1	Highest in Southern Asia	Autosomal recessive
*TFR2*	Chromosome 7	Highest among Non-Finnish Europeans	Autosomal recessive
*HAMP*	Chromosome 19	Several populations	Autosomal recessive
*SLC40A1*	Chromosome 2	Several populations, highest among African populations	Autosomal dominant

**Table 2:** Iron content in selected food sources and preparations comparing both animal and plant-sourced, per 100 g, ranked from most to least. Information from U.S. Department of Agriculture’s Food Data Central database.

**Animal-Source (Heme)**	**Iron (mg) per 100 g**
Chicken, liver, simmered	11.6
Oyster, moist heat	9.21
Mussels, moist heat	6.72
Beef, liver, braised	6.54
Beef, tenderloin, roast, choice, roasted	3.11
Sardines, canned in oil	2.92
Duck, meat, roasted	2.7
Lamb, sirloin chop, broiled	2.34
Beef, ground, 90% lean meat	2.3
Turkey, ground, cooked	1.52
Turkey, dark meat, roasted	1.43
Tuna, light, canned in oil	1.39
Egg, whole, scrambled	1.31
Pork, tenderloin, roasted	1.15
Salmon, Atlantic wild, dry heat	1.03
**Plant-Source (Non-heme)**	**Iron (mg) per 100 g**
Tofu, raw, regular	5.36
Soybeans, boiled	5.14
Molasses	4.72
Bread, wheat, toasted	4.09
Pistachios, raw	3.92
Lentils, boiled	3.33
Red kidney beans, boiled	2.94
Walnuts, raw	2.91
Chickpeas, boiled	2.89
Spinach, raw	2.71
Pecans, raw	2.51
Lima beans, boiled	2.39
Black beans, boiled	2.1
Pinto beans, boiled	2.09
Pasta, whole-wheat, cooked	1.72

**Table 3:** Most common formulations and elemental iron content in oral ferrous iron supplements. Adapted from Ning and Zeller, 2019 [58].

Compound	Formulation	Elemental Iron Content (mg)	Recommended Dosage
Ferrous gluconate	Tablet, 240 mg	27	1–3 tablets once per day or on an alternate-day schedule [58]
Ferrous sulfate	Tablet, 325 mg	65	1–2 tablets once per day or on an alternate-day schedule [58]
Ferrous fumarate	Tablet, 324 mg	106	1 tablet once per day or on an alternate-day schedule [58]

**Table 4:** Most common formulations, recommended dosages, and infusion times of intravenous iron therapies. The list in this table is not exhaustive. Adapted from Ning and Zeller, 2019 [58].

Form	Amount per Dose Recommended (mg)	Infusion Time	Reference
Iron dextran (Low-molecular-weight)	100	2 to 6 h	[93]
Ferrous gluconate	125	12.5 mg per min	[94]
Iron sucrose	200–300	100 mg per 5 min	[95]
Ferric carboxymaltose	750	15 min	[96]

## Data Availability

Not applicable.
